# Robot-assisted laparoscopic hepatectomy for hepatocellular carcinoma with Fontan-associated liver disease: a world-first case report

**DOI:** 10.1186/s40792-024-02014-5

**Published:** 2024-09-09

**Authors:** Takuma Ishikawa, Shinji Itoh, Takeo Toshima, Shohei Yoshiya, Yuki Bekki, Norifumi Iseda, Yuriko Tsutsui, Ichiro Sakamoto, Kotaro Abe, Tomoharu Yoshizumi

**Affiliations:** 1 Department of Surgery and Science, Graduate School of Medical Sciences, Kyushu University, 3-1 Maidashi, Higashi-Ku, Fukuoka-Shi, Fukuoka, 812-0054 Japan; 2 Department of Cardiovascular Medicine, Graduate School of Medical Sciences, Kyushu University, Fukuoka, Japan

**Keywords:** Fontan procedure, Fontan-associated liver disease, Robot-assisted laparoscopic hepatectomy, Hepatocellular carcinoma, Central venous pressure

## Abstract

**Background:**

Fontan-associated liver disease (FALD) encompasses hepatic complications following the Fontan procedure, ranging from fibrosis to hepatocellular carcinoma (HCC). Despite advancements in surgical techniques and perioperative care, robot-assisted laparoscopic hepatectomy (RALH) for HCC in patients with FALD has not been previously reported owing to concerns about the Fontan circulation.

**Case presentation:**

We present the first case of RALH for recurrent HCC in a 45-year-old man after the Fontan procedure. The preoperative evaluation confirmed good cardiac function. The procedure involved meticulous monitoring and management of central venous pressure and was successfully completed with minimal blood loss. Postoperative recovery was uneventful. With thorough preoperative cardiac assessment and close collaboration between cardiologists and anesthesiologists, RALH can be safely performed in selected patients with FALD.

**Conclusions:**

Even if a patient has a history of FALD, RALH can be safely performed in selected patients under appropriate conditions.

## Background

Liver complications after undergoing the Fontan procedure (FP) are classified as Fontan-associated liver disease (FALD) [1]. The first report of FALD was by Stanton et al. in 1981 when cirrhosis was found in an autopsy case who died of a fatal arrhythmia 21 months after the FP [2]. FALD is a severe complication, particularly in adult patients who have undergone the FP [3]. FALD encompasses a range of hepatic abnormalities, from mild fibrosis to cirrhosis and hepatocellular carcinoma (HCC), and is associated with benign and malignant liver lesions [4]. The pathophysiology of FALD is complex, involving hemodynamic and inflammatory factors [3]. Recently, the long-term prognosis after the FP has been improving owing to advances in procedures and postoperative management [5]. As a result, the number of patients diagnosed with FALD, a late complication of liver fibrosis and cirrhosis leading to HCC, is increasing.

Robot-assisted surgery is rapidly gaining popularity in many surgical fields, because it is precise, minimally invasive surgery with enhanced stereoscopic magnification enabling full dexterity [6]. The first report of robot-assisted laparoscopic hepatectomy (RALH) in the world was made by Giulianotti in 2003 [7]. Several limitations and drawbacks to conventional laparoscopy exist, including limited movement, the inability to perform high-precision sutures, unnatural positions for the surgeon, and flat vision. Robotic surgery can overcome the limitations of conventional laparoscopy. The goal is for this type of minimally invasive surgery to be available to more patients [7].

Despite the known benefits of RALH, its results have never been reported after the FP. The limitations of RALH in FALD are related primarily to the adverse effects of pneumoperitoneum on the Fontan circulation due to increasing intra-abdominal and intrathoracic pressure, rising pulmonary and systemic resistance, and cardiac preload and output reduction, which can be fatal [8]. Moreover, severe portal hypertension may cause a high risk of bleeding during liver resection [9]. To the best of our knowledge, this report is the first case of RALH for HCC after the FP.

### Case presentation

We present the case of a 45-year-old man with HCC recurrence. The patient had a history of tricuspid atresia and underwent a Blalock–Taussig–Thomas shunt procedure at the age of 3, an FP at the age of 6, and total cavopulmonary bypass conversion, atrial septal defect creation, right atrium maze procedure, and implantation of a permanent pacemaker lead at the age of 26. At age 42, the patient had elevated alpha-fetoprotein levels, and a detailed examination revealed HCC. Lap-assisted partial resection of segment 3 of the liver was performed. Pathological findings indicated a moderate to poorly differentiated HCC with trabecular and pseudoglandular patterns of 2.5 × 1.9 cm. This was classified as vp1, vv1, va0, b0, im0, and the peritumoral liver tissue showed stage F3 cirrhosis according to the new Inuyama classification [10]. No malignant cells were found in the surgical margins (R0 resection).

**Fig. 1:**
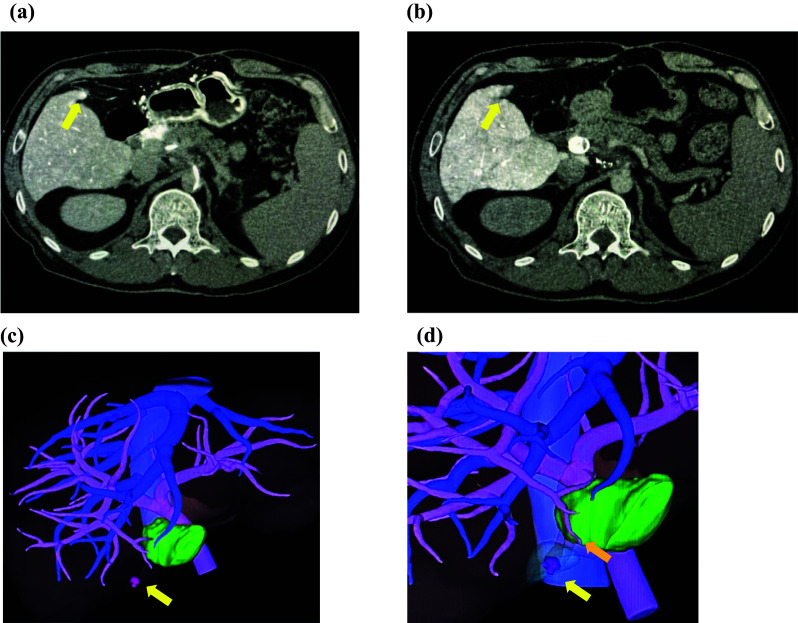
Abdominal computed tomography arteriography and arterial portography. **a** 8 mm arterially enhanced nodule with corona-like enhancement was observed in segment 5 of the liver (yellow arrowhead). **b** Same nodule exhibited poor portal blood flow (yellow arrowhead). **c** Position of the tumor is indicated in the 3D images (yellow arrowhead). **d** 3D images show that the tumor was located on the edge of segment 5 (yellow arrowhead) and branch of the Glisson 5 (orange arrowhead)

During follow-up, HCC recurrence was suspected in segment 5 of the liver on computed tomography (CT) 25 months postoperatively. CT hepatic arteriography/CT arterial portography revealed a reticular pattern of decreased enhancement in the hepatic parenchyma, indicative of changes due to congestive liver. An 8 mm arterially enhanced nodule was observed with corona-like enhancement in segment 5 of the liver **(**Fig. 1a**)** and a lack of portal blood flow, consistent with findings of HCC **(**Fig. 1b**).** The tumor was located on the edge of segment 5, and the nearest branch of the Glisson 5 was not within the planned resection margin **(**Fig. 1c, d**).**

Preoperative information included a height of 164 cm and a weight of 57.1 kg (body mass index, 21.1). Blood testing showed the following results: aspartate transaminase, 35 U/L; alanine transaminase, 56 U/L; albumin, 4.2 mg/dL; total bilirubin, 0.9 mg/ dL; prothrombin time-international normalized ratio, 1.08; and platelet count, 12.8 × 104/μL. Alpha-fetoprotein and des-gamma-carboxy prothrombin were elevated to 11.1 ng/mL and 7538 mAU/mL, respectively. Type IV collagen 7S was slightly elevated to 6.0 ng/mL, but other markers of liver fibrosis were normal (hyaluronic acid, 39 ng/mL; mac-2-binding protein glycosylation isomer, 0.34 cutoff index). Negative results were obtained for hepatitis B virus surface antigen and hepatitis C virus antibody, and the patient had no history of heavy alcohol consumption. The indocyanine green (ICG) retention rate at 15 min was 15.9%. The Child–Pugh classification was A. Echocardiography demonstrated good single right ventricular function and no obstruction in the Fontan circulation. Cardiac function tests showed an ejection fraction of 61%, central venous pressure (CVP) of 10–14 mmHg, pulmonary artery pressure of 17 mmHg, and pulmonary capillary wedge pressure of 11 mmHg. Pulmonary and renal functions were normal. Oxygen saturation in room air was 96%. The systolic blood pressure was approximately 90 mmHg.

Robot-assisted laparoscopic partial resection of S5 was planned to address the recurrent HCC after a multidisciplinary discussion with the cardiologist and anesthesiologist. Warfarin 2 mg was discontinued and replaced with heparin. Two days before the surgery, 12.5 mg of ICG was administered intravenously to the patient. After induction of general anesthesia, a central venous catheter was inserted into the right internal jugular vein for intraoperative monitoring of CVP. A transesophageal echocardiogram was also placed. Management was performed by monitoring cardiac output and its variations using a FloTrac sensor^®^. The cardiovascular medications used in this case were dobutamine, vasopressin, furosemide, ephedrine, and phenylephrine.

### Surgical procedure

**Fig. 2:**
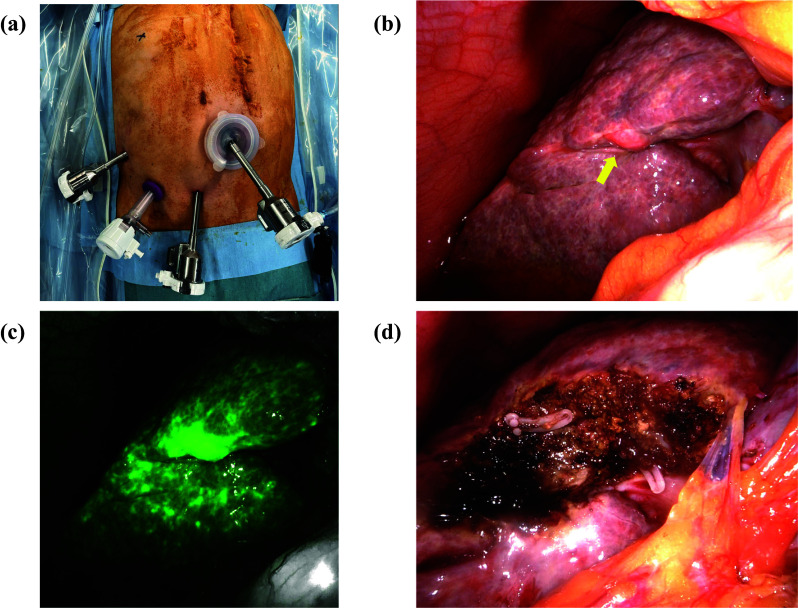
Operative findings. **a** Surgery was performed by the insertion of four ports: one subumbilical for the camera (8 mm), one working trocar in the right lateral flank (8 mm), one working trocar in the lower right abdomen position (8 mm), and one assistant port between the second and third ports (12 mm). **b** Lliver showed a rough surface consistent with cirrhosis. The tumor in S5 was visibly protruding (yellow arrowhead). **c** Tumor in S5 was revealed using indocyanine green fluorescence for confirmation. **d** Robot-assisted laparoscopic S5 partial hepatectomy resections were performed

The patient was placed in the supine position. A small incision was made in the subumbilical area, and an access port was placed to induce pneumoperitoneum. A pneumoperitoneum was started at a pressure of 8 mmHg while carefully monitoring vital signs. The liver showed a rough surface consistent with cirrhosis, and a small amount of ascites was observed. Adhesions between the abdominal wall and omentum were noted. The lesion in S5 was visibly protruding **(**Fig. 2a**)**. The second port was placed 20 cm away from the tumor, and the third port was positioned 7 cm away in the lower right abdomen. An assistant port (12 mm) was placed 7 cm away between the second and third ports. To add additional ports, it was necessary to perform adhesiolysis within the abdominal cavity. Adequate visualization of the tumor had been achieved, and it was deemed feasible to resect the marginal tumor with four ports. We performed intraperitoneal manipulation using four ports and a minimal number of instruments. **(**Fig. 2b**)**.

**Figure. 3.**
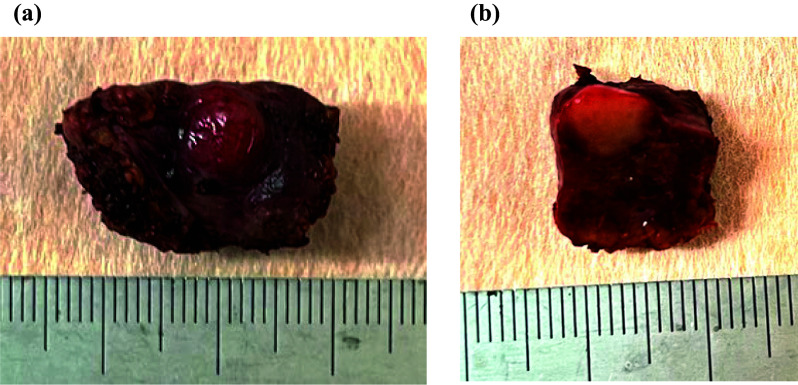
Macroscopic findings of the resected tissue specimen. **a** Elastic soft nodule was found in liver segment 5. **b** On the cut surface, there was a yellowish-white tumor measuring 0.8 cm × 0.8 cm

The patient cart was rolled in and docked from the right side. The falciform ligament was not divided. Adhesions were dissected to allow for liver resection. Adhesions around the hepatoduodenal ligament were observed. Furthermore, since the lesion was small and located on the surface of segment 5, we refrained from forcibly Pringle maneuver. The resection line was set to ensure adequate margins. Although the gallbladder was located near the tumor, we were able to secure clear resection margins for the tumor, thus preserving the gallbladder. Liver parenchymal transection was performed using the crush-clamp technique with intraoperative ICG fluorescence to confirm the tumor margins (Fig. 2c). Small, exposed vessels on the transection surface were coagulated and divided as necessary. The specimen was extracted (Fig. 2d). Intra-abdominal lavage was performed, and hemostasis was confirmed. A 15 Fr drain was placed through the 8 mm port on the right side of the abdomen. The robotic docking was released, and the patient cart was rolled out (console time was 54 min). The specimen was extracted through the umbilical incision. After confirming the tumor was at a sufficient distance from the resection margin, the incision was closed **(**Fig. 3**)**. The operation duration was 111 min, with minimal blood loss.

### Histological findings

A well to moderately differentiated HCC with compact, trabecular, and pseudoglandular patterns of 0.6 × 0.6 cm was classified as vp1, vv0, va0, b0, im0. The peritumoral liver tissue showed stage F4 cirrhosis according to the new Inuyama classification [10] No malignant cells were found in the surgical margins (R0 resection).

### Postoperative course

Postoperatively, the patient was managed in the intensive care unit. Owing to low blood pressure, norepinephrine was administered. On the first postoperative day, the drain was removed as its condition was unremarkable. Norepinephrine was discontinued, and the patient was transferred to the general ward and began oral intake. On the second postoperative day, heparin therapy was initiated. On the third postoperative day, oral warfarin was resumed. CT on the sixth postoperative day revealed no issues. Heparin therapy was discontinued on the seventh postoperative day. The patient was discharged on the eighth postoperative day. One year after surgery, the patient has not had HCC recurrence.

## Discussion

We presented a case of robotic-assisted laparoscopic liver resection in a patient with FALD where reoperation was necessary due to HCC recurrence. No reports of robotic-assisted laparoscopic liver resection for HCC with FALD exist. Liver dysfunction arising from FALD causes liver fibrosis, cirrhosis, and HCC, even in young patients [11]FALD is caused by excessive hepatic congestion due to high CVP and results from fibrosis of the sinusoids and portal tracts. The progression of FALD depends on the duration after the FP and hepatic venous pressure [12]. It has been reported that FALD progresses to cirrhosis 11–15 years after the FP, with cumulative incidence rates of cirrhosis of 56.6% and 97.9% at 20 and 30 year post-FP, respectively [13]. The annual incidence of HCC in FALD is estimated to be between 1.5% and 5.0% [14].

Many cases of HCC arising from FALD are treated non-surgically because of poor liver function, making surgical resection unfeasible [9]]. However, recent advancements in surgical techniques and perioperative management have made safe hepatectomy possible in open and laparoscopic surgery [14, 15] Laparoscopic hepatectomy for HCC arising from FALD has often been avoided owing to the difficulty in controlling venous bleeding caused by high CVP and challenges in anesthesia management [16].

Robot-assisted laparoscopic surgery in patients with Fontan circulation is considered disadvantageous, because the venous return may be compromised by insufflation of carbon dioxide (CO₂) into the abdomen, use of the reverse Trendelenburg position, and positive pressure ventilation [14, 15, 17]. Furthermore, CO₂ absorption from the peritoneum increases the partial pressure of CO₂ in the blood, raising pulmonary vascular resistance. CO₂ in the abdominal cavity might be sucked into the injured hepatic veins, causing pulmonary embolism. The Pringle maneuver does not adversely affect the Fontan circulation during laparoscopic hepatectomy [14]. On the other hand, inferior vena cava clamping can easily induce hypotension in the Fontan circulation [5]. In this case, the patient was placed in the reverse Trendelenburg position, and the CVP was controlled at approximately 10 mmHg during the surgery. The preoperative CVP in the previous surgery was 19 mmHg, and the CVP was controlled at approximately 15 mmHg during the surgery. In the current surgery, the patient was managed by the cardiology department preoperatively, with the preoperative CVP ranging from 10 to 14 mmHg and the intraoperative CVP controlled at 10 mmHg. In case of FALD, minimally invasive surgery, particularly RALH, was considered candidate only when the lesion was small, located on the surface of anterolateral segments, and did not involve major blood vessels, and the Fontan circulation was maintained despite a low CVP. Furthermore, this case involved reoperation, and compared to laparoscopic surgery, the advantages of RALH were considered beneficial for performing precise tasks, such as adhesiolysis, in confined spaces. However, a disadvantage of RALH was the time required to convert to open surgery, which was crucial in cases involving FALD. The indication for RALH in patients with FALD must be carefully determined through close communication with cardiologists and anesthesiologists.

RALH has several disadvantages compared with conventional laparoscopic hepatectomy, including prolonged operation time and increased total cost [18, 19]. However, RALH offers several advantages over conventional laparoscopic hepatectomy, including enhanced visualization, improved surgical dexterity, ease of dissection and suturing, and stable camera control. [20, 21]. Comparisons between robotic and laparoscopic hepatectomies indicate that both methods are equally safe and feasible in terms of blood loss, transfusion rates, and postoperative complications [22]. Nevertheless, robotic surgery has a higher rate of performing major hepatectomies in a minimally invasive manner [22]. In addition, during robotic-assisted laparoscopic surgery, the timing of conversion to open surgery must always be considered because of the risk of intraoperative bleeding and the likelihood of vital sign changes in patients with FALD. Therefore, hepatectomy for HCC arising from FALD requires stricter criteria than conventional hepatectomy. Such criteria may include preoperative CVP to predict the likelihood of intraoperative bleeding from hepatic veins. To establish such criteria, data should be accumulated on patients with FALD by creating a large-scale, nationwide database. In this surgery, the tumor was located on the liver surface; therefore, we were able to perform the hepatectomy relatively easily. In the future, reports on hepatectomy for tumors in the deep liver parenchyma will also be necessary.

In conclusion, our report suggests that RALH for patients with HCC and Fontan circulation can be safely performed in selected patients with sufficient cardiac reserves. A long time has passed since the FP was first performed, and the cases of HCC with congested liver cirrhosis are expected to increase in the future. In addition, because of congestive cirrhosis and carcinogenesis at a young age, recurrence of HCC may be unavoidable. RALH is a desirable minimally invasive surgery for reoperation and maintaining postoperative quality of life. More case reports on RALH for patients with HCC and Fontan circulation are warranted.

## Conclusion

We successfully performed RALH in a patient with FALD. To safely perform RALH for HCC in patients who have undergone the FP, thorough preoperative cardiac function assessments, careful decision-making, and close communication between cardiologists and anesthesiologists are necessary.

## Data Availability

The datasets used and/or analysed during the current study are available from the corresponding author on reasonable request.

## Abbreviations

CO₂: Carbon dioxide
CT: Computed tomography
CVP: Central venous pressure
FALD: Fontan-associated liver disease
FP: Fontan procedure
HCC: Hepatocellular carcinoma
ICG: Indocyanine green
RALH: Robot-assisted laparoscopic hepatectomy
